# Analyzing the immunogenicity of bivalent booster vaccinations in healthcare workers: The SWITCH ON trial protocol

**DOI:** 10.3389/fimmu.2022.1067749

**Published:** 2022-11-29

**Authors:** Ngoc H. Tan, Roos S. G. Sablerolles, Wim J. R. Rietdijk, Abraham Goorhuis, Douwe F. Postma, Leo G. Visser, Susanne Bogers, Daryl Geers, Luca M. Zaeck, Marion P. G. Koopmans, Virgil A. S. H. Dalm, Neeltje A. Kootstra, Anke L. W. Huckriede, Debbie van Baarle, Melvin Lafeber, Corine H. GeurtsvanKessel, Rory D. de Vries, Paul-Hugo Marie van der Kuy

**Affiliations:** ^1^ Department of Hospital Pharmacy, Erasmus Medical Center, Rotterdam, Netherlands; ^2^ Center of Tropical Medicine and Travel Medicine, Department of Infectious Diseases, Amsterdam University Medical Centers, Amsterdam, Netherlands; ^3^ Infection & Immunity, Amsterdam Public Health, University of Amsterdam, Amsterdam, Netherlands; ^4^ Department of Internal Medicine and Infectious Diseases, University Medical Center Groningen, Groningen, Netherlands; ^5^ Department of Infectious Diseases, Leiden University Medical Center, Leiden, Netherlands; ^6^ Department of Viroscience, Erasmus Medical Center, Rotterdam, Netherlands; ^7^ Department of Internal Medicine, Division of Allergy & Clinical Immunology and Department of Immunology, Erasmus Medical Center, Rotterdam, Netherlands; ^8^ Department of Experimental Immunology, Amsterdam University Medical Centers, Amsterdam Infection and Immunity Institute, University of Amsterdam, Amsterdam, Netherlands; ^9^ Department of Medical Microbiology and Infection Prevention, University Medical Center Groningen, University of Groningen, Groningen, Netherlands; ^10^ Center for Infectious Disease Control, National Institute for Public Health and the Environment, Bilthoven, Netherlands; ^11^ Department of Internal Medicine, Erasmus Medical Center, Rotterdam, Netherlands

**Keywords:** COVID-19, immune memory, mRNA vaccine, adenovirus-based vaccine, immune response

## Abstract

Vaccination against coronavirus disease 2019 (COVID-19) has contributed greatly to providing protection against severe disease, thereby reducing hospital admissions and deaths. Several studies have reported reduction in vaccine effectiveness over time against the Omicron sub-lineages. However, the willingness to receive regular booster doses in the general population is declining. To determine the need for repeated booster vaccinations in healthy individuals and to aid policymakers in future public health interventions for COVID-19, we aim to gain insight into the immunogenicity of the additional bivalent booster vaccination in a representative sample of the healthy Dutch population. The SWITCH ON study was initiated to investigate three main topics: i) immunogenicity of bivalent vaccines after priming with adenovirus- or mRNA-based vaccines, ii) immunological recall responses and reactivity with relevant variants after booster vaccination, and iii) the necessity of booster vaccinations for the healthy population in the future.

**Clinical trial registration:**

https://clinicaltrials.gov/, identifier NCT05471440.

## Background

Vaccination against coronavirus disease 2019 (COVID-19) has contributed greatly to providing protection against severe disease, thereby reducing hospital admissions and deaths (1–3). However, the emergence of the antigenically distinct Omicron sub-lineages, combined with waning antibody levels after vaccination, once again put pressure on public health, healthcare services and the economy of many countries at the end of 2021 (4). The mutated receptor binding domain (RBD) of the spike (S) protein allows Omicron sub-lineages to spread more efficiently and enhances antibody evasion (5–7). Several studies have reported reduction in vaccine-induced neutralizing antibodies cross-reactive with Omicron sub-lineages (8, 9), although T cell immunity remains largely intact (8, 10, 11). Severe acute respiratory distress syndrome coronavirus-2 (SARS-CoV-2) breakthrough infections can occur even after receiving a booster vaccination, or after acquiring infection-induced immune responses. Recent observational studies showed a significant reduction in vaccine effectiveness against the Omicron sub-lineages over time, even after a booster dose (12–14). As a result, there is an increased demand for updated vaccines that can provide better protection against emerging variants.

Bivalent vaccines that contain both the ancestral S protein, to boost previously induced immune responses, and the Omicron BA.1 or BA.5 S protein, to specifically boost antibodies recognizing viruses from the Omicron sub-lineage, have been designed for that reason. A study on one bivalent vaccine reported superiority in the induction of neutralizing antibodies to omicron sub-lineage variant BA.1 compared to the previously approved monovalent vaccine (15). Although the seasonality of SARS-CoV-2 outbreaks has not been determined yet and varies across different climates, more frequent surges have been observed in colder months (16). In preparation for the winter and probable circulation of a SARS-CoV-2 variant from the Omicron sub-lineage, bivalent vaccines have been made rapidly available for upcoming vaccination campaigns.

We now know that sterile immunity to SARS-CoV-2 infection is not achieved by vaccination, and that SARS-CoV-2 continues to circulate among humans. However, booster vaccinations remain important to prevent severe disease, especially in immunocompromised risk groups and the elderly. The willingness to receive regular booster doses in the general population is declining, partly because of the reduced disease severity observed after the emergence of the Omicron sub-lineages. Therefore, questions have arisen about future vaccination policies. Over 70% of the European adult population has completed the basic series of COVID-19 vaccination, yet just over 50% has received an additional booster dose (17). To determine the need for repeated booster vaccinations in healthy individuals and to aid policymakers in future public health interventions for COVID-19, we aim to gain insight into the immunogenicity of the additional bivalent booster vaccination in a representative sample of the healthy Dutch population. The SWITCH ON study was initiated to investigate three main topics: i) immunogenicity of bivalent vaccines after priming with adenovirus- or mRNA-based vaccines, ii) immunological recall responses and reactivity with relevant variants after booster vaccination, and iii) the necessity of booster vaccinations for the healthy population in the future.

## SWITCH ON study design

SWITCH ON is a multi-center, open labelled, randomized, controlled trial. A total of 400 participants, between 18 and 65 years old, will be recruited from healthcare workers across four university medical centers (UMC) in the Netherlands (i.e., Amsterdam UMC, Erasmus MC Rotterdam, UMC Groningen, and Leiden UMC). Participants will be predominantly, but not exclusively, recruited from previously published SWITCH (9, 18–20) and healthcare worker (HCW) studies (7, 8, 21). Extensive records on immune response to vaccinations against COVID-19 and breakthrough infections are available for participants from those two studies. Prior infection with SARS-CoV-2 is accepted, but not in the three months before the start of the study. Some additional exclusion criteria will be applied, such as pregnancy, immunosuppressive medication consumption, receiving cancer therapy, or allergic reactions to ingredients of the bivalent vaccine. A full overview of inclusion and exclusion criteria can be found in **Supplementary File 1**, page 17. Half of the participants of SWITCH ON were primed with an adeno-based vaccine (Ad26.COV2.S), while the other half were primed with an mRNA-based vaccine (either BNT162b2 or mRNA-1273). The trial starts recruiting in September 2022, vaccinations will start in October 2022.

**Figure 1:**
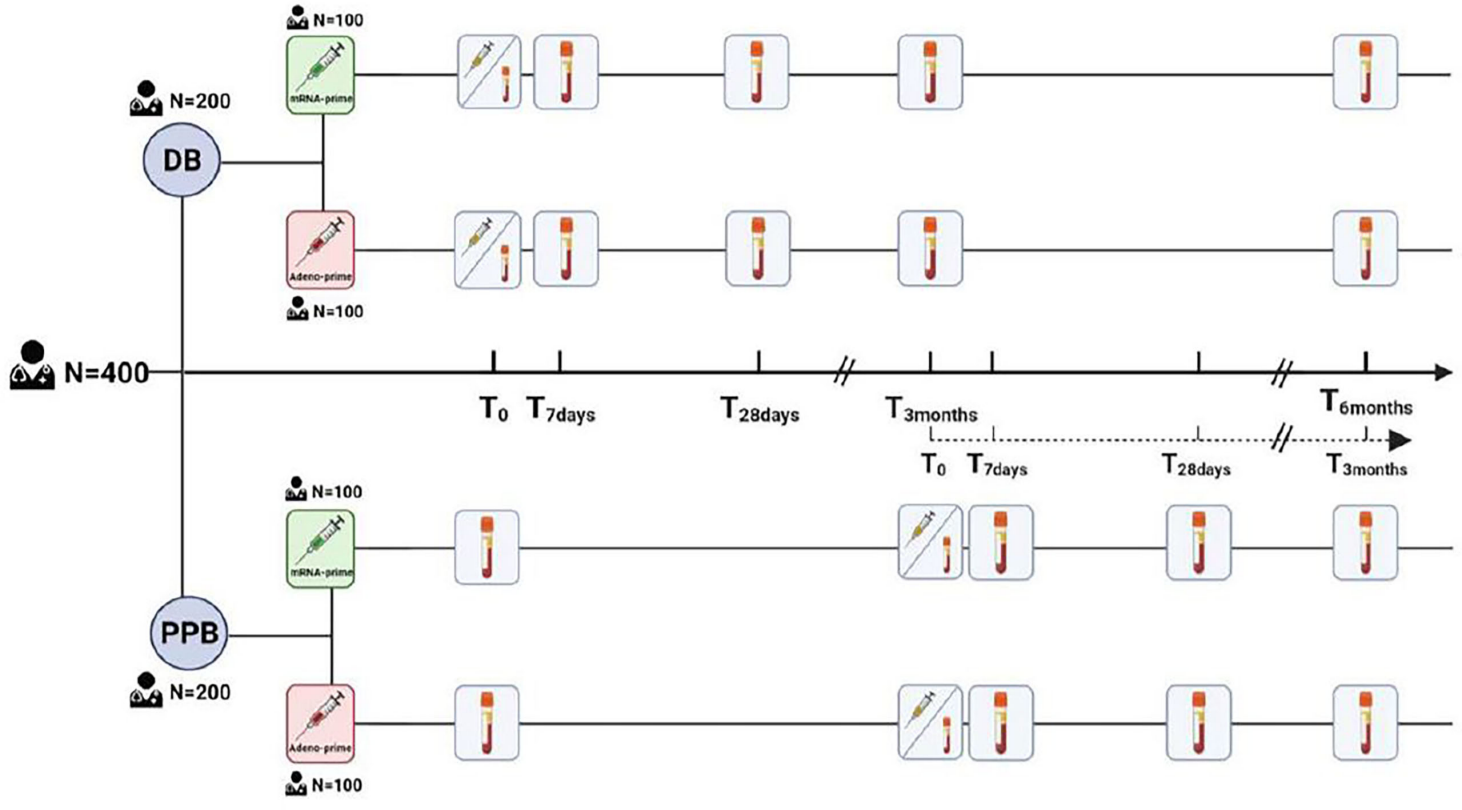
The 400 participants will be split into two groups, each consisting of 100 participants primed with Ad26.COV2.S (red) and 100 participants primed with an mRNA-based vaccine (green). The immunological response will be measured on day 0, 7, 28, and at 3 and 6 months after vaccination.

Participants will be equally divided into two study arms: direct boost (DB) or postponed boost (PPB). The DB arm consists of two branches: one with 100 Ad26.COV2.S-primed participants and the other with 100 mRNA-based-primed participants. The PPB arm has the same structure (**Figure 1**). We refer to **Supplementary File 1**, page 18, for the sample size calculation. Participants in the DB arm will receive the bivalent (ancestral/OMI BA.1) booster dose in the first week of October 2022, as per Dutch policy. Participants in the PPB group will receive the booster 3 months later than those in the DB arm; it is expected that by that time the novel bivalent ancestral/BA.5 vaccines will be used. Block randomization with 1:1 ratio was used between DB and PPB groups with stratification for priming vaccine type. By randomizing over the different groups, we assume that prior infections are equally distributed. Participants will be asked by questionnaire about prior COVID-19 (whether and when a participant had a prior SARS-CoV-2 infection). To confirm prior infection, or to identify missed sub-clinical COVID-19 cases, N-specific antibodies will be retrospectively measured in the pre-boost sample from all participants. A record of breakthrough infections after booster vaccination will be compiled *via* questionnaires and retrospective assessment of N-specific antibodies in serum collected 3 months after booster vaccination. To measure vaccine immunogenicity, SARS-CoV-2 specific antibody and T-cell responses will be measured in all participants at day 0, 7 and 28, and 3 months after booster administration. Neutralizing antibodies against variants encoded by the vaccine (ancestral SARS-CoV-2, and the Omicron BA.1 and BA.5 variants), and circulating variants (e.g. Omicron BQ.1.1 and XBB) will be measured by an in-house developed plaque reduction neutralization test (PRNT) in a random selection of participants. The PRNT has been extensively validated but is RUO as there is no comparable IVD registered alternative. SARS-CoV-2-specific T-cell responses will be assessed *via* two assays (1): a CE-IVD whole-blood interferon gamma release assay (IGRA, QuantiFERON SARS-CoV-2, QIAGEN) on all participants, and (2) a RUO activation induced marker (AIM) flow cytometry assay. This assay will use overlapping peptide pools from the ancestral S protein, and the Omicron BA.1, and BA.5 S proteins to stimulate peripheral blood mononuclear cells (PBMCs) isolated from 25% of participants in each branch of the trial arms. Cryopreserved PBMCs will additionally be used for the quantification and phenotyping of virus-specific B-cells, by staining with the ancestral, BA.1, or BA.5 S protein. The data will be analyzed on an intention-to-treat basis.

## Outcome

The primary outcome is defined as the fold-change in SARS-CoV-2-specific binding antibodies between day 0 and 28 for the adenovirus-primed and mRNA-based-primed participants, measured by quantitative IgG assay with the ancestral and Omicron BA.1 S protein. The secondary outcome focuses on recall responses induced by booster vaccination. To this end, S-specific antibodies and T-cells will be measured and compared on day 7 and 28 post boost. Clinical trials involving bivalent vaccines thus far only measured neutralizing antibodies at day 28 after the booster (15, 22). The analysis of the response at day 7 has two advantages: i) rapid recall responses after vaccination could be a proxy for recall upon infection, indicative of whether booster vaccinations are required or not, and ii) early immunogenicity evaluation can accelerate the interpretation of future studies. Finally, the breadth of immunological responses will be studied in depth by comparing antibody and T-cell reactivity to the relevant SARS-CoV-2 variants (similar to our previous study (20)). With respect to safety, we will analyze the reactogenicity during the first 7 days after booster vaccination. We strive to publish the results as soon as possible to inform the policymakers and aid individual decision making regarding the booster dose.

## Implications

The COVID-19 pandemic is currently in a transition phase towards endemic circulation, with reduced morbidity and mortality at the population level compared to the initial waves of circulation. Given the growing societal aversion towards repeated booster vaccinations, an important question is whether, and for how long, time-intervals between subsequent booster vaccinations can be prolonged. Differences in recall responses depending on the initial priming schedule (mRNA-based or vector-based) can guide the direction of future booster strategies, whereas the speed and breath of recall cellular memory after booster vaccination will provide important information for the timing of future booster vaccinations. The impact of intercurrent COVID-19 infections despite earlier vaccinations needs to be factored in when assessing these immune responses.

## Data availability statement

The raw data supporting the conclusions of this article will be made available by the authors, without undue reservation.

## Ethics statement

The studies involving human participants were reviewed and approved by METC Erasmus MC. The patients/participants provided their written informed consent to participate in this study. Written informed consent was obtained from the individual(s) for the publication of any potentially identifiable images or data included in this article.

## Author contributions

All authors were involved in the design and execution of the study and writing of the manuscript. All authors contributed to the article and approved the submitted version.

## Funding

The trial is funded by Netherlands Organization for Health Research and Development ZonMw in the COVID-19 Vaccine program (project grant number: 10430072110001).

## Conflict of interest

The authors declare that the research was conducted in the absence of any commercial or financial relationships that could be construed as a potential conflict of interest.

## Publisher’s note

All claims expressed in this article are solely those of the authors and do not necessarily represent those of their affiliated organizations, or those of the publisher, the editors and the reviewers. Any product that may be evaluated in this article, or claim that may be made by its manufacturer, is not guaranteed or endorsed by the publisher.
