# Supplementary material for: Analyzing the immunogenicity of bivalent booster vaccinations in healthcare workers: The SWITCH ON trial protocol
Source: Front Immunol. 2022 Nov 29;13:1067749. doi: 10.3389/fimmu.2022.1067749 (PMC9744953; doi:10.3389/fimmu.2022.1067749)
Supplement: Supplementary file 1 [file DataSheet_1.pdf]

SWITCH

SWITCH

SWITCH ON:

Analysing the immunogenicity of additional booster vaccinations in healthcare workers.

A multicenter, randomised, controlled trial

SWITCH

Protocol ID	METC-nummer: MEC-2022-0462<br>ABR-nummer: NL81983.078.22<br>Panama nummer: 9170<br>Clinicaltrial.gov: NCT05471440
**Short title**	SWITCH ON
**EudraCT number**	**2022-002560-73**
**Version**	1.2
**Date**	31-08-2022
**Coordinating investigator/project leader**	Drs. Roos Sablerolles<br>Dr. Virgil Dalm<br>Dr. Corine Geurts van Kessel<br>Dr. Rory de Vries
**Principal investigator(s) (in Dutch: hoofdonderzoeker/ uitvoerder)**	**Erasmus MC**<br>Prof. dr. Hugo van der Kuy<br>Dr. Wim Rietdijk<br>Dr. Melvin Lafeber<br>Prof. dr. Marion Koopmans<br><br>**UMCG**<br>Prof. dr. Debbie van Baarle<br>Prof. dr. Anke Huckriede<br>Dr. Douwe Postma<br><br>**AmsterdamUMC**<br>Dr. Bram Goorhuis<br>Dr. Neeltje Kootstra<br><br>**LUMC**<br>Prof. dr. Leo Visser

**Sponsor (in Dutch: verrichter/opdrachtgever)**	Erasmus MC Rotterdam, the Netherlands
**Subsidising party**	ZonMw (request is being processed)
**Independent expert (s)**	Dr. N.C. Peltenburg<br>n.c.peltenburg@erasmusmc.nl
**Laboratory sites <*if applicable*>**	Viroscience laboratory, Erasmus MC, the Netherlands<br><br>Biochemistry laboratory, Erasmus MC, the Netherlands
**Pharmacy <*if applicable*>**	Hospital Pharmacy, Erasmus MC, The Netherlands

PROTOCOL SIGNATURE SHEET

Name	Signature	Date
**Sponsor or legal representative:**<br>Prof. dr. Hugo van der Kuy<br>Hospital Pharmacist/Clinical Pharmacologist<br>Head of Hospital Pharmacy	[signature]	31-08-2022
**Coordinating Investigator/Project leader/Principal Investigator:**<br><br>Dr. Virgil Dalm<br>internist-clinical immunologist	[signature]	31-08-2022

SWITCH

TABLE OF CONTENTS

1. INTRODUCTION AND RATIONALE 12
2. OBJECTIVES 14
3. STUDY DESIGN 16
4. STUDY POPULATION 17
4.1 Population (base) 17
4.2 Inclusion criteria 17
4.3 Exclusion criteria 17
4.4 Sample size calculation 17
5. TREATMENT OF SUBJECTS 19
5.1 Investigational product/treatment 19
5.2 Use of co-intervention (if applicable) 19
5.3 Escape medication (if applicable) 19
6. INVESTIGATIONAL PRODUCT 20
6.1 Name and description of non-investigational product(s) 20
6.2 Summary of findings from non-clinical studies 20
6.3 Summary of findings from clinical studies 20
6.4 Summary of known and potential risks and benefits 21
6.5 Description and justification of route of administration and dosage 22
6.6 Dosages, dosage modifications and method of administration 22
6.7 Preparation and labelling of Non Investigational Medicinal Product 22
6.8 Drug accountability 22
7. NON-INVESTIGATIONAL PRODUCT 23
7.1 Name and description of non-investigational product(s) 23
7.2 Summary of findings from non-clinical studies 23
7.3 Summary of findings from clinical studies 23
7.4 Summary of known and potential risks and benefits 23
7.5 Description and justification of route of administration and dosage 23
7.6 Dosages, dosage modifications and method of administration 23
7.7 Preparation and labelling of Non Investigational Medicinal Product 23
7.8 Drug accountability 23
8. METHODS 24
8.1 Study parameters/endpoints 24
8.1.1 Main study parameter/endpoint 24
8.1.2 Secondary study parameters/endpoints 24
8.1.3 Exploratory objective 25
8.2 Randomisation, blinding and treatment allocation 25
8.3 Study procedures 26
8.4 Withdrawal of individual subjects 28
8.4.1 Specific criteria for withdrawal (if applicable) 28
8.5 Replacement of individual subjects after withdrawal 28
8.6 Follow-up of subjects withdrawn from treatment 28

8.7 Premature termination of the study ... 28
9. SAFETY REPORTING ... 29
9.1 Temporary halt for reasons of subject safety ... 29
9.2 AEs, SAEs and SUSARs ... 29
9.2.1 Adverse events (Aes) ... 29
9.2.2 Serious adverse events (SAEs) ... 29
9.2.3 Suspected unexpected serious adverse reactions (SUSARs) ... 30
9.3 Annual safety report ... 31
9.4 Follow-up of adverse events ... 31
9.5 [Data Safety Monitoring Board (DSMB) / Safety Committee] ... 31
10. STATISTICAL ANALYSIS ... 33
10.1 Primary study parameter(s) ... 33
10.2 Secondary study parameter(s) ... 34
10.3 Other study parameters ... 34
10.4 Interim analysis (if applicable) ... 34
11. ETHICAL CONSIDERATIONS ... 35
11.1 Regulation statement ... 35
11.2 Recruitment and consent ... 35
11.3 Objection by minors or incapacitated subjects (if applicable) ... 36
11.4 Benefits and risks assessment, group relatedness ... 36
11.5 Compensation for injury ... 37
11.6 Incentives (if applicable) ... 37
12. ADMINISTRATIVE ASPECTS, MONITORING AND PUBLICATION ... 38
12.1 Handling and storage of data and documents ... 38
i. Confidentiality of the data ... 38
ii. Confidentiality of subject records ... 38
iii. Data Management ... 38
iv. Biological samples ... 39
12.2 Monitoring and Quality Assurance ... 39
12.3 Amendments ... 39
12.4 Annual progress report ... 39
12.5 Temporary halt and (prematurely) end of study report ... 39
12.6 Public disclosure and publication policy ... 40
13. STRUCTURED RISK - BENEFIT ANALYSIS ... 41
13.1 Potential issues of concern ... 41
13.2 Synthesis ... 41
Benefits and risks assessment, group relatedness ... 41
14. REFERENCES ... 42

LIST OF ABBREVIATIONS AND RELEVANT DEFINITIONS

ABR	General Assessment and Registration form (ABR form), the application form that is required for submission to the accredited Ethics Committee; in Dutch: Algemeen Beoordelings- en Registratieformulier (ABR-formulier)
AE	Adverse Event
AR	Adverse Reaction
CA	Competent Authority
CCMO	Central Committee on Research Involving Human Subjects; in Dutch: Centrale Commissie Mensgebonden Onderzoek
CV	Curriculum Vitae
DSMB	Data Safety Monitoring Board
EU	European Union
EudraCT	European drug regulatory affairs Clinical Trials
GCP	Good Clinical Practice
GDPR	General Data Protection Regulation; in Dutch: Algemene Verordening Gegevensbescherming (AVG)
HCW	HealthCare Workers
IB	Investigator's Brochure
IC	Informed Consent
IMP	Investigational Medicinal Product
IMPD	Investigational Medicinal Product Dossier
METC	Medical research ethics committee (MREC); in Dutch: medisch-ethische toetsingscommissie (METC)
PRNT	Plaque Reduction Neutralization Test
(S)AE	(Serious) Adverse Event
SPC	Summary of Product Characteristics; in Dutch: officiële productinformatie IB1-tekst
Sponsor	The sponsor is the party that commissions the organisation or performance of the research, for example a pharmaceutical company, academic hospital, scientific organisation or investigator
SUSAR	Suspected Unexpected Serious Adverse Reaction
UAVG	Dutch Act on Implementation of the General Data Protection Regulation; in Dutch: Uitvoeringswet AVG
WMO	Medical Research Involving Human Subjects Act; in Dutch: Wet Medisch-wetenschappelijk Onderzoek met Mensen

SUMMARY

Rationale:
Eighty percent of the Dutch population has completed a primary COVID-19 vaccination regimen, and 60% of the population received a booster vaccination. Waning immunity, combined with the emergence of antigenically distinct SARS-CoV-2 variants, has led to the consideration of additional booster vaccinations in the Dutch population by autumn 2022. However, despite efforts of the Dutch policymakers, the public's willingness to repeatedly receive COVID-19 booster vaccinations is declining. This is mainly due to a reduced burden of disease by COVID-19, fewer hospitalizations, and fewer deaths. However, population immunity might be one of the major factors responsible for this reduced burden of disease, possibly emphasizing the need for booster vaccinations. In this proposal we will address an important question asked by policymakers: **“Are booster vaccinations in autumn recommended for the healthy population?”**

In the SWITCH ON study we propose to address this question by performing a multicenter, randomized, controlled trial investigating the immunogenicity of an additional COVID-19 booster vaccination in healthcare workers (HCW). Here, we will answer two sub-questions.

1. Is there an increase in antibody levels between day of boost and 28 days after boosting HCW that were initially primed with either the Janssen or an mRNA-based vaccine? This information obtained in a representative sample will be directly shared with the policymakers to inform decision-making for additional booster vaccinations.
2. Does booster vaccination lead to a rapid recall response, indicative of immunological memory? The presence of a rapid recall response could inform policymakers that the Dutch population is still readily ‘boost-able’, and that booster vaccinations are not yet warranted.

Since the combined answer to the two sub-questions could indicate that booster vaccinations in autumn are not yet warranted, policymakers will ask the question when booster vaccinations are warranted. To this end, we propose to perform the booster vaccinations in two separate phases: 1) as soon as possible (Direct Boost - DB), and 2) 3-4 months from start of the study (PostPoned Boost - PPB).

Our study cohort will consist of both Janssen-primed and mRNA-primed (Moderna or Pfizer) HCW. The participants of the original SWITCH study (1-3), a unique and well-defined cohort, will be invited for this follow-up study. Additionally, we will invite participants of the Erasmus MC HCW study (4, 5) that received an mRNA-based priming vaccination. These individuals were primed with Moderna or Pfizer, samples were obtained before prime, 28 days after first vaccination, and 28 days and 6 months after the second vaccination. Additional samples were obtained before booster vaccination with an mRNA-based vaccine, and 28 days and 6 months after boost. This makes the Erasmus MC HCW cohort equally well-defined as the SWITCH cohort. By combining Janssen and mRNA-based primed HCW in this study, we make the proposed study representative for the Dutch population.

To provide supportive data for policymakers, we will administer a (to be determined) booster vaccination to the above-described cohort before additional booster vaccination is implemented in society (DB).

The PPB group will be used to investigate the effect of postponing a boost with 3 / 4 months on immune response and can be used to advice the policymakers if the national vaccination campaign is postponed for healthy individuals. To get comparable groups between DB and PPB all participants are randomized at the start of the study and blood will be drawn for all participants at the first study visit.

At this time, the primary endpoint used in most vaccination trials is 28 days post-boost. However, we propose to additionally measure immune responses 7 days post-boost in this study for 2 important reasons:

1. It is known that immunological memory remains intact longer than antibodies can be measured in the periphery, and that this immunological memory is responsible for rapid recall responses upon re-exposure. Measuring immune responses as early as 7 days post-boost is a good alternative measure for functional immunological memory. The presence of boost-able memory may be a supportive argument for the reasoning that booster vaccination is not (yet) necessary.
2. To reduce the time to provide data to policymakers. We propose to measure both binding antibody levels and T-cell responses at day 7 and 28, and to analyse whether the levels on day 28 can be predicted on day 7. If so, the information from the proposed trial could fast-track the interpretation of future studies by 3 weeks.

Objective: The key objective of the study to determine whether it is **recommended to boost the healthy population in autumn.**

Study design: A multicenter, randomized, controlled trial comparing immune responses 7 and 28 days after an additional COVID-19 booster vaccination between Janssen and mRNA primed HCWs to describe the immune response in a cohort representative of the Dutch population, in order to eventually provide data for Dutch policy makers.
Setting: multicenter study conducted through four university hospitals (i.e, Amsterdam UMC, Erasmus MC, Leiden UMC, and UMC Groningen).

Study population: Participants will be recruited from both the SWITCH-trial (Janssen primed HCWs) (1-3) and the Erasmus MC HCW-study (mRNA primed HCWs) (4, 5), as these participants are well characterized and documented in earlier responses to COVID-19 vaccinations. Participants may participate regardless of a previous SARS-CoV-2 infection. By randomizing over the different groups, we assume that the experienced infections are equally distributed over the groups. To assess whether this is truly the case, N-specific antibodies will be retrospectively measured in the pre-boost sample from all participants.

Intervention (if applicable): Participants will be randomised into a DB or a PPB group after stratification for priming (mRNA versus Janssen). The immune response will be measured at start of the study (first study visit, all participants) and 0, 7, 28 and 84 days after boost.

Main study parameters/endpoints:
<u>Primary endpoint</u>:
The primary endpoint is to determine the fold change in antibody level at day of boost and 28 days after an additional COVID-19 booster vaccination in the direct boost group, comparing Janssen with mRNA-primed HCW.

To specifically answer the key objective, we have defined two questions with the following outcome parameters:

1. *Is there an increase in antibody levels between day of boost and 28 days after boosting HCW that were initially primed with either the Janssen or an mRNA-based vaccine?*
   Outcome: Level and fold change of antibodies determined by a quantitative IgG assay comparing the Janssen primed and mRNA-based primed HCW.

2. *Does booster vaccination lead to a rapid recall response, indicative of immunological memory?*
   Outcome: Level and fold change of antibodies and T-cell responses determined by a quantitative IgG assay and whole blood IFNγ release assay, respectively, comparing day 7 and 28 post-boost.

Depending on whether it is possible to conduct this study with a bivalent vaccine, we will add an Omicron BA.1 IgG test to the arsenal of analyses.

<u>Secondary endpoints:</u>
In addition to the primary endpoints defined above, this study will address the following secondary objectives:

1. *What is the difference in booster immunogenicity comparing a direct boost with a postponed boost?*
   Outcome: Level of antibodies and T-cell responses 7 and 28 days post boost in DB versus PPB group.

2. *What is the breadth of the immune responses after booster vaccination?*
   Outcome: Plaque Reduction Neutralization Test (PRNT) against relevant variants in a random selection of study participants.

3. *What is the predictive value of immune responses on day 7 post boost?*
   Outcome: Relationship between antibodies and T-cell responses on day 7 and 28 post boost.

4. *What is the difference in reactogenicity 7 days after boost comparing the Janssen and mRNA primed HCW?*
   Outcome: Adverse events (AE) first 7 days after an additional boost between Janssen and mRNA primed HCW.

5. *Initial examination of breakthrough infections before and during study period.*
   Outcome: Database of breakthrough infections in included participants based on positive PCR, self-reported positive antigen test, or detection of N-specific antibodies.

Nature and extent of the burden and risks associated with participation, benefit and group relatedness: The consequence of participating in this study has two sides; on the one hand, a higher burden for the participant, given 5 study visits in one year including blood samples and questionnaires. It should be mentioned that complications such as pain from

drawing blood or, for example, a bruise can arise. In addition, side effects of vaccination can arise. On the other hand, participants receive an overview of their own immune response after vaccination at several time points and their participation contributes to answering a very relevant research question. We expect the burden of the study visits to be minimal.

1. INTRODUCTION AND RATIONALE

Eighty percent of the Dutch population has completed a primary COVID-19 vaccination regimen, and 60% of the population received a booster vaccination. Waning immunity, combined with the emergence of antigenically distinct SARS-CoV-2 variants, has led to the consideration of additional booster vaccinations in the Dutch population by autumn 2022. However, despite efforts of the Dutch policymakers, the public's willingness to repeatedly receive COVID-19 booster vaccinations is declining. This is mainly due to a reduced burden of disease by COVID-19, fewer hospitalizations, and fewer deaths. However, population immunity might be one of the major factors responsible for this reduced burden of disease, possibly emphasizing the need for booster vaccinations. In this proposal we will address an important question asked by policymakers: **“Are booster vaccinations in autumn recommended for the healthy population?”**

In the SWITCH ON study we propose to address this question by performing a multicenter, randomized, controlled trial investigating the immunogenicity of an additional COVID-19 booster vaccination in HCW. Here, we will answer two sub-questions.

1. *Is there an increase in antibody levels between day of boost and 28 days after boosting HCW that were initially primed with either the Janssen or an mRNA-based vaccine?* This information obtained in a representative sample will be directly shared with the policymakers to inform decision-making for additional booster vaccinations.
2. *Does booster vaccination lead to a rapid recall response, indicative of immunological memory?* The presence of a rapid recall response could inform policymakers that the Dutch population is still readily ‘boostable’, and that booster vaccinations are not yet warranted.

Since the combined answer to the two sub-questions could indicate that booster vaccinations in autumn are not yet warranted, policymakers will ask the question when booster vaccinations are warranted. To this end, we propose to perform the booster vaccinations in two separate phases: 1) as soon as possible (Direct Boost - DB), and 2) 3-4 months from start of the study (PostPoned Boost - PPB).

Our study cohort will consist of both Janssen-primed and mRNA-primed (Moderna or Pfizer) HCW. The participants of the original SWITCH study (1-3), a unique and well-defined cohort, will be invited for this follow-up study. Additionally, we will invite participants of the Erasmus MC HCW study (4, 5) that received an mRNA-based priming vaccination. These individuals were primed with Moderna or Pfizer, samples were obtained before prime, 28 days after first vaccination, and 28 days and 6 months after the second vaccination. Additional samples were obtained before booster vaccination with and mRNA-based vaccine, and 28 days and 6 months after boost. This makes the Erasmus MC HCW cohort equally well-defined as the SWITCH cohort. By combining Janssen and mRNA-based primed HCW in this study, we make the proposed study representative for the Dutch population.

To provide supportive data for policymakers, we will administer a *(to be determined)* booster vaccination to the above-described cohort before additional booster vaccination is implemented in society (DB).

The PPB group will be used to investigate the effect of postponing a boost with 3 / 4 months on immune response and can be used to advice the policymakers if the national vaccination campaign is postponed for healthy individuals. To get comparable groups between DB and PPB all participants are randomized at the start of the study and blood will be drawn for all participants at the first study visit.

At this time, the primary endpoint used in most vaccination trials is 28 days post-boost. However, we propose to additionally measure immune responses 7 days post boost in this study for 2 important reasons:

1. It is known that immunological memory remains intact longer than antibodies can be measured in the periphery, and that this immunological memory is responsible for rapid recall responses upon re-exposure. Measuring immune responses as early as 7 days post-boost is a good alternative measure for functional immunological memory. The presence of boostable memory may be a supportive argument for the reasoning that booster vaccination is not (yet) necessary.
2. To reduce the time to provide data to policymakers. We propose to measure both binding antibody levels and T-cell responses at day 7 and 28, and to analyse whether the levels on day 28 can be predicted on day 7. If so, the information from the proposed trial could fast-track the interpretation of future studies by 3 weeks.

2. OBJECTIVES

Key objective

The key objective of the study to determine whether it is **recommended to boost the healthy population in autumn.**

Primary objective

Primary endpoint:

The primary endpoint is to determine the fold change in antibody level at day of boost and 28 days after an additional COVID-19 booster vaccination in the direct boost group, comparing Janssen with mRNA-primed HCW.

To specifically answer the key objective, we have defined two questions with the following outcome parameters:

1. *Is there an increase in antibody levels between day of boost and 28 days after boosting HCW that were initially primed with either the Janssen or an mRNA-based vaccine?*
   Outcome: level and fold change of antibodies determined by a quantitative IgG assay comparing the Janssen primed and mRNA-based primed HCW.

2. *Does booster vaccination lead to a rapid secondary recall response, indicative of immunological memory?*
   Outcome: Level and fold change of antibodies and T-cell responses determined by a quantitative IgG assay and whole blood IFNγ release assay, respectively, comparing day 7 and 28 post boost.

Depending on whether it is possible to conduct this study with a bivalent vaccine, we will add an Omicron BA.1 IgG test to the arsenal of analyses.

Secondary objectives

In addition to the primary endpoints defined above, this study will address the following secondary objectives:

1. *What is the difference in booster immunogenicity comparing a direct boost with a postponed boost?*
   Outcome: Level of antibodies and T-cell responses 7 and 28 days post boost in DB versus PPB group.

2. *What is the breadth of the immune responses after booster vaccination?*
   Outcome: PRNT against relevant variants in a random selection of study participants.

3. *What is the predictive value of immune responses on day 7 post boost?*
   Outcome: Relationship between antibodies and T-cell responses on day 7 and 28 post boost.

4. *What is the difference in reactogenicity 7 days after boost comparing the Janssen and mRNA primed HCW?*
   Outcome: Adverse events (AE) first 7 days after an additional boost between Janssen and mRNA primed HCW.

5. *Initial examination of breakthrough infections before and during study period.*
   Outcome: Database of breakthrough infections in included participants based on positive PCR, self-reported positive lateral flow test, or detection of N-specific antibodies.

Exploratory objectives

Exploratory objectives (dependent on vaccine type and analyzed outside requested funding):

- Gene expression profiles associated with recall response (PAXgene tube)
- SARS-CoV-2-specific T-cell responses (PBMC, assessed by activation-induced marker assay and / or TCRbeta sequencing.

3. STUDY DESIGN

A multicenter, randomized, controlled trial comparing immune responses 7 and 28 days after an additional COVID-19 booster vaccination between Janssen and mRNA primed HCWs to describe the immune response in a cohort representative of the Dutch population, in order to eventually provide data for Dutch policy makers.

Setting
Multicenter study conducted through four university hospitals (i.e, Amsterdam UMC, Erasmus MC, Leiden UMC, and UMC Groningen).

Trial duration
Total duration of each participant will be 6/7 months from the administration of the additional boost.

Study groups
This study will consists of 2 cohorts; SWITCH study (Janssen prime - from four university hospitals; Amsterdam UMC, Erasmus MC, Leiden UMC, and UMC Groningen) and HCW study (mRNA prime from Erasmus MC) (n=200 per cohort).

Group	Intervention*	Number of participants	Neutralization (3 variants)
DB mRNA	Boost	100	15
DB Janssen	Boost	100	15
PPB mRNA	Boost	100	15
PPB Janssen	Boost	100	15

*****_Which vaccine will be administered as intervention remains to be determined, based on availability._

Randomisation/Blinding
Participants will be stratified by Janssen or mRNA prime and randomised 1:1 fashion (DB versus PPB) using block randomisation.
Participants of this study will be blinded to their first study visit. At the time of the first study visit, they will be told whether they will be vaccinated in the DB or PPB. Blood will be drawn from all participants at the first study visit.
All participants randomized to the DB group will receive the same vaccine at the first study visit. All participants randomized to the PPB group will receive their vaccine 3 / 4 months later.
Vaccines will be prepared in the hospital pharmacy. Eight days after the boost, they will be asked to complete the side effects questionnaires for 7 days after the boost and blood will be drawn at all given time points.

4. STUDY POPULATION

4.1 Population (base)

Healthcare Workers (HCW) from 18 to 65 years old who were primed with one Janssen or two mRNA vaccines (Moderna or Pfizer). Individuals of all ethnicities will be recruited. Participants will be recruited from both the SWITCH-trial (Janssen primed HCWs) (1-3) and the Erasmus MC HCW-study (mRNA primed HCWs) (4, 5), as these participants are well characterized and documented in breakthrough infections and earlier responses to COVID-19 vaccinations. Participants may participate regardless of a previous SARS-CoV-2 infection(s). By randomizing over the different groups, we assume that the experienced infections are equally distributed over the groups. To assess whether this is truly the case, N-specific antibodies will be retrospectively measured in the pre-boost sample from all participants.

4.2 Inclusion criteria

1. Participant is willing and able to give written informed consent for participation in the trial.
2. Adult (male/female) between 18 and 65 years old
3. Sufficient level of the Dutch language to undertake all study requirements

4.3 Exclusion criteria

1. Adults younger than 18 or older than 65 years.
2. Adults primed with another vaccine than Janssen, Moderna or Pfizer.
3. History of allergic reactions likely to be exacerbated by any component of study vaccines (e.g. hypersensitivity to the active substance or any of the SmPC-listed ingredients of the Janssen/Pfizer/Moderna vaccine).
4. Adults that are pregnant.
5. Currently being treated for cancer.
6. Severe kidney failure or dialyses dependent.
7. Status after organ-, stem cell- or bone marrow transplantation.
8. Use of immunosuppressant's.
9. Epilepsy.
10. HIV.
11. Bleeding disorder (e.g. factor deficiency, coagulopathy or platelet disorder), or prior history of significant bleeding of bruising following IM injections of vene puncture.
12. Continuous use of anticoagulants, such as coumarins (e.g. acenocoumarol) or novel oral anticoagulants (i.e. apixaban, dabigatran etc).
13. Participants who are currently participating in another research trial.
14. All regular contra-indications of the vaccines will be applied.

4.4 Sample size calculation

The primary analysis of this study will consist of one comparison:

- The fold change (day of boost and day 28) in antibody response after an additional boost comparing Janssen and mRNA primed HCW in the direct boost arm.

The sample size calculation is based on the following assumptions:

- The sample size calculation takes the available data from the Erasmus MC HCW study.
- The log10 transformed difference in fold change 28 days after third vaccination (i.e., second booster after Janssen prime and first boost after mRNA prime) are *Janssen prime 1.344 (sd=0.451);*
  mRNA prime 1.151 (sd=0.449).
- The expected difference is estimated using the non-parametric Mann-Whitney U test (mean and standard deviations are used to estimated median and interquartile ranges). This procedure is readily available in G*Power (version 3.1.9.7).

Based on the above assumptions, the study needs to recruit 91 patients in each arm to achieve 80% power at two-sided 5% significance level. We assume +/- 10% of study participants will be loss to follow-up. Therefore, the sample size in each arm will be 100 participants. The study contains two treatment arms and two groups resulting in a total sample size of 400 participants.

5. TREATMENT OF SUBJECTS

Vaccines against SARS-CoV-2 (wildtype) are standard of care in the Netherlands. This study will investigate the immune response and AEs after an additional boost in autumn 2022. Which vaccine will be used will depend on the advice of ZonMw and the RIVM. It could be the regular vaccine from Moderna or Pfizer, but also the new variant vaccine from Moderna. This depends on the speed at which the new vaccine is registered by the European Medicines Agency. If the HCW are not likely to participate in this study, they will not yet receive a booster vaccine and have to wait what the government policy will be.

5.1 Investigational product/treatment

All participants are vaccinated with the same vaccine. It is yet to be determined which vaccine will be used. The vaccine is given into the muscle of the upper arm. The vaccines will come directly from RIVM and are not part of the stock used for the national vaccination program. These vaccinations are reserved by RIVM for research purposes. All used vaccines are registered COVID-19 Vaccines.

5.2 Use of co-intervention (if applicable)

Not applicable

5.3 Escape medication (if applicable)

Not applicable

6. INVESTIGATIONAL PRODUCT

The vaccines that are used are officially registered by the EMA. It is yet to be determined which vaccine will be used. This will be advised by RIVM and OMT at the end of July.

Vaccine	Registration date + Link SmPC
Pfizer	23/12/2021<br>Clean COVID-19 Vaccine SmPC-PL 21-DEC-2020 735AM EST-135PM CET (europa.eu)
Moderna	06/01/2021<br>COVID-19 Vaccine Moderna, Common name-COVID-19 mRNA Vaccine (nucleoside modified) (europa.eu)

6.1 Name and description of non-investigational product(s)

Vaccine	Description
Pfizer	Comirnaty contains a molecule called messenger RNA (mRNA) with instructions for producing a protein from SARS-CoV-2, the virus that causes COVID-19. Comirnaty does not contain the virus itself and cannot cause COVID-19.
Moderna	Moderna contains a molecule called messenger RNA (mRNA) with instructions for producing a protein from SARS-CoV-2, the virus that causes COVID-19. Comirnaty does not contain the virus itself and cannot cause COVID-19.

6.2 Summary of findings from non-clinical studies

Not applicable. The vaccine is registered.

6.3 Summary of findings from clinical studies

Vaccine	Clinical findings
Pfizer	43.548 patients<br>A two-dose regimen of BNT162b2 conferred 95% protection against Covid-19 in persons 16 years of age or older. Safety over a median of 2 months was similar to that of other viral vaccines.<br>Safety and Efficacy of the BNT162b2 mRNA Covid-19 Vaccine - PubMed (nih.gov)
Moderna	30.000 patients<br>Conclusions: The mRNA-1273 vaccine showed 94.1% efficacy at

	preventing Covid-19 illness, including severe disease. Aside from transient local and systemic reactions, no safety concerns were identified.<br>Efficacy and Safety of the mRNA-1273 SARS-CoV-2 Vaccine - PubMed (nih.gov)

6.4 Summary of known and potential risks and benefits

Potential risks from vaccine administration (Moderna / Pfizer).
Hypersensitivity to the active substance or to any of the excipients.
The most common side effects are usually mild or moderate and got better within a few days after vaccination. Local and systemic adverse reactions were reported more frequently after dose 2 than after dose 1.
Very common (>10%): pain and/or swelling at the injection site, lymphadenopathy (axillary on the same side as the injection site), headache, nausea, vomiting, muscle and joint pain, fatigue, chills, and fever.
Common (1-10%): (itchy)rash or redness at the injection side. Skin rash.
Uncommon (0.1-1%): itching at the injection side, insomnia, malaise, pain in extremities, tremor, sneezing, sore throat, hyperhidrosis, muscle weakness, backache, and asthenia.
Rare (0.01-0.1%): hypersensitivity (allergic reaction of skin and underlying tissue), urticaria. Acute peripheral facial paralysis or palsy (in reports seen from day 22 to day 32 after the 2nd dose with Moderna and from day 3 to 48 after vaccination with Pfizer.
Facial swelling reported in individuals who have had facial dermal fillers in the past (Moderna).
Very rare (<0.01%): thrombosis in combination with thrombocytopenia; includes venous thrombosis such as cerebral venous sinus thrombosis, splanchnic venous thrombosis and arterial thrombosis.
Furthermore, anaphylaxis and hypersensitivity have been reported.
As for all vaccines, the vaccines will be given under close supervision with appropriate medical treatment available.

Potential benefits
These vaccines offer a high level of protection against COVID-19 which is a critical need in the current pandemic (see table for efficacy rates). Most side effects are mild to moderate in severity and are gone within a few days. The European Medicines Agency therefore decided that these vaccine benefits are greater than its risks and that they can be authorized for use in the EU. All vaccines have been granted a conditional marketing authorization. This means that there is more evidence to come about the vaccine, which the company is required to provide. The European Medicines Agency will review any new information that becomes available and this overview will be updated as necessary. However, the evidence is overwhelming that these vaccines at least prevent hospitalizations from COVID.

Vaccine	Efficacy (depended on variant)
Pfizer	up to 95%
Moderna	up to 95%

6.5 Description and justification of route of administration and dosage

All vaccines will be administered according to their respectively SmPC registration.
Also the dosage will be administered according to the SmPC.
All vaccines are given into the muscle of the upper arm.

6.6 Dosages, dosage modifications and method of administration

Vaccine	Dosage
Pfizer	0.3 ml IM after dilution with NaCl 0.9%
Moderna	0.25 ml IM

6.7 Preparation and labelling of Non Investigational Medicinal Product

Vaccine	Preparation
Pfizer	The thawed vaccine must be diluted in its original vial with 1.8 mL sodium chloride 9 mg/mL (0.9%) solution for injection, using a 21 gauge or narrower needle and aseptic techniques.<br>Gently invert the diluted dispersion 10 times. Do not shake.<br>Withdraw 0.3 mL of Comirnaty.
Moderna	Withdraw 0.25 mL of Moderna.

6.8 Drug accountability

The vaccines will be supplied by the RIVM after approval by ZonMw and Ministry of Health, wellbeing and sports (VWS). It is important to state that the vaccines for this study will NOT be retrieved from a stock reserved for patients or other persons.

As is usual in routine care at this moment, the batch numbers will be recorded for each patients ensuring its traceability. Vaccines will be shipped to the pharmacy. A drug accountability form will be used to record IMP dispensing.

7. NON-INVESTIGATIONAL PRODUCT

7.1 Name and description of non-investigational product(s)

Not applicable

7.2 Summary of findings from non-clinical studies

Not applicable

7.3 Summary of findings from clinical studies

Not applicable

7.4 Summary of known and potential risks and benefits

Not applicable

7.5 Description and justification of route of administration and dosage

Not applicable

7.6 Dosages, dosage modifications and method of administration

Not applicable

7.7 Preparation and labelling of Non Investigational Medicinal Product

Not applicable

7.8 Drug accountability

Not applicable

This paragraph is not applicable. Only vaccines will be used described in paragraph 6.

8. METHODS

8.1 Study parameters/endpoints

8.1.1 Main study parameter/endpoint

The primary endpoint is to determine the fold change in antibody level at day of boost and 28 days after an additional COVID-19 booster vaccination in the direct boost group, comparing Janssen with mRNA-primed HCW.

To specifically answer the key objective, we have defined two questions with the following outcome parameters:

1) *Is there an increase in antibody levels between day of boost and 28 days after boosting HCW that were initially primed with either the Janssen or an mRNA-based vaccine?*
Outcome: Level and fold change of antibodies determined by a quantitative IgG assay comparing the Janssen primed and mRNA-based primed HCW.
2) *Does booster vaccination lead to a rapid secondary recall response, indicative of immunological memory?*
Outcome: Level and fold change of antibodies and T-cell responses determined by a quantitative IgG assay and whole blood IFNγ release assay, respectively, comparing day 7 and 28 post-boost.

Depending on whether it is possible to conduct this study with a bivalent vaccine, we will add an Omicron BA.1 IgG test to the arsenal of analyses.

8.1.2 Secondary study parameters/endpoints

<u>Secondary endpoints:</u>
In addition to the primary endpoints defined above, this study will address the following secondary objectives:

1. *What is the difference in booster immunogenicity comparing a direct boost with a postponed boost?*
   Outcome: Level of antibodies and T-cell responses 7 and 28 days post boost in DB versus PPB group.

2. *What is the breadth of the immune responses after booster vaccination?*
   Outcome: PRNT against relevant variants in a random selection of study participants.

3. *What is the predictive value of immune responses on day 7 post boost?*
   Outcome: Relationship between antibodies and T-cell responses on day 7 and 28 post boost.

4. *What is the difference in reactogenicity 7 days after boost comparing the Janssen and mRNA primed HCW?*
   Outcome: Adverse events (AE) first 7 days after an additional boost between Janssen and mRNA primed HCW.

5. *Initial examination of breakthrough infections before and during study period*
   Outcome: Database of breakthrough infections in included participants based on positive PCR, self-reported positive lateral flow test, or detection of N-specific antibodies.

8.1.3 **Exploratory objective**

<u>Other study parameters (if applicable)</u>
For the analysis of the data the study parameters which are collected are gender, age, co-medication, comorbidity, vaccination status and actual (gone through) COVID-19 infections.

8.2 Randomisation, blinding and treatment allocation

The cohort will be filled by Amsterdam UMC (n=40), LUMC (n=40), UMCG (n=40) and Erasmus MC (n=280) with 400 participants vaccinated primarily with Janssen (n=200) or mRNA (n=200; Moderna/Pfizer). Participants will be randomised in one of the following groups, stratified by priming vaccine.

- Direct boost
- Postponed boost

Randomization will be done after obtaining written informed consent.

SAMPLING STRATEGY

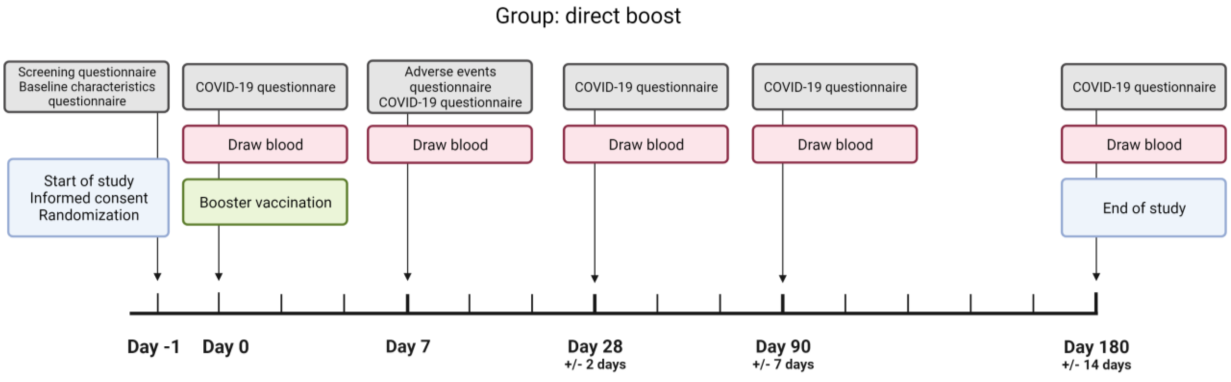

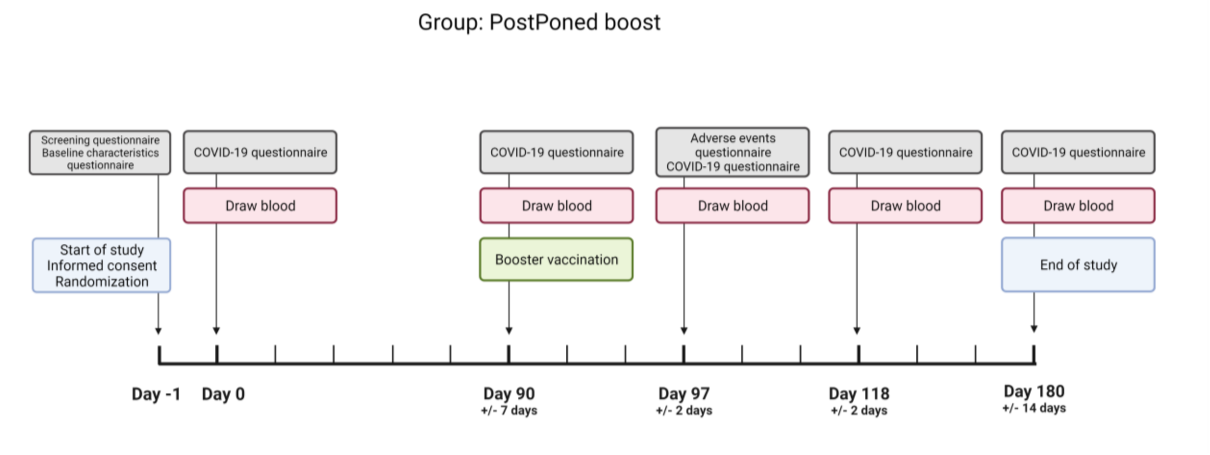

8.3 Study procedures

All participants at day of informed consent

- Sign informed consent
- Randomization in DB or PPB group
- Fill a baseline characteristics questionnaire

All participants at day 0:

DB group

- Request to fill a COVID-19 questionnaire (online questionnaires via e-mail).
- One serum tube (8.5ml)
- One lithium heparin tube (10ml)
- Four lithium heparin tubes (4x10ml)*
- Boost with additional vaccine

*only in the detailed immunological assessment group

PPB group

- Request to fill a COVID-19 questionnaire (online questionnaires via e-mail).
- One serum tube (8.5ml)
- One lithium heparin tube (10ml)
- Four lithium heparin tubes (4x10ml)*

*only in the detailed immunological assessment group

All participants DB group after 7 days (day 7)

- Request to fill a side effects + COVID-19 questionnaire (solicited local & systemic reactions)
- One serum tube (8.5ml)
- One lithium heparin tube (10ml)
- Four lithium heparin tubes (4x10ml)*

*only in the detailed immunological assessment group

All participants DB group after 28 days (day 28)

- Request to fill a COVID-19 questionnaire
- One serum tube (4ml)

- One lithium heparin tube (10ml)
- Four lithium heparin tubes (4x10ml)*

*only in the detailed immunological assessment group

All participants DB group after 180 days (day 180)

- Request to fill a COVID-19 questionnaire
- One serum tube (4ml)
- One lithium heparin tube (10ml)
- Four lithium heparin tubes (4x10ml)*

*only in the detailed immunological assessment group

All participants PPB group after 90 days (day 90) or 112 days (day 112)

- Request to fill a COVID-19 questionnaire (online questionnaires via e-mail).
- One serum tube (8.5ml)
- One lithium heparin tube (10ml)
- Four lithium heparin tubes (4x10ml)*
- Boost with additional vaccine

*only in the detailed immunological assessment group

All participants PPB group after 97 days (day 97) or 119 days (day 119)

- Request to fill a COVID-19 questionnaire
- One serum tube (4ml)
- One lithium heparin tube (10ml)
- Four lithium heparin tubes (4x10ml)*

*only in the detailed immunological assessment group

All participants PPB group after 118 days (day 118) or 140 days (day 140)

- Request to fill a COVID-19 questionnaire
- One serum tube (4ml)
- One lithium heparin tube (10ml)
- Four lithium heparin tubes (4x10ml)*

*only in the detailed immunological assessment group

All participants PPB group after 180 days (day 180) or 202 days

- Request to fill a COVID-19 questionnaire
- One serum tube (4ml)
- One lithium heparin tube (10ml)
- Four lithium heparin tubes (4x10ml)*

*only in the detailed immunological assessment group

<u>Questionnaires</u>

Prior to the study, all participants will be given a screening questionnaire to determine whether they are eligible to participate or not. This, because a lot can have changed in the health situation in one year.
At the start of the study, a questionnaire about the basic characteristics of the HCW follows. Seven days after boost one questionnaire will be used to monitor side effects of

the vaccine. At each visit one additional questionnaire will be used to monitor occurrence and outcome of COVID-19 infection throughout the study.

8.4 Withdrawal of individual subjects

Subjects can leave the study at any time for any reason if they wish to do so without any consequences. The investigator can decide to withdraw a subject from the study for urgent medical reasons.

8.4.1 Specific criteria for withdrawal (if applicable)

Not applicable

8.5 Replacement of individual subjects after withdrawal

With the sample size calculation we have taken into account 10% lost to follow up. We will not add participants if participants drop out / quit the study.

8.6 Follow-up of subjects withdrawn from treatment

Participants who withdraw from the study will receive the same standard treatment as described in the then applicable guidelines. Already collected and distributed samples/ specimens and data will not be destroyed or deleted.

8.7 Premature termination of the study

If there is a danger to participants as a result of an additional boost, all necessary steps will be taken to safeguard the safety of the participants.

9. SAFETY REPORTING

9.1 Temporary halt for reasons of subject safety

In accordance to section 10, subsection 4, of the WMO, the sponsor will suspend the study if there is sufficient ground that continuation of the study will jeopardise subject health or safety. The sponsor will notify the accredited METC without undue delay of a temporary halt including the reason for such an action. The study will be suspended pending a further positive decision by the accredited METC. The investigator will take care that all subjects are kept informed.

9.2 AEs, SAEs and SUSARs

9.2.1 Adverse events (Aes)

Adverse events are defined as any undesirable experience occurring to a subject during the study, whether or not considered related to the trial procedure. All adverse events reported spontaneously by the subject or observed by the investigator or his staff will be recorded. However, given the setting of a large outbreak, the registration of Aes is not feasible and will not serve the safety of the participants. Therefore, Aes will not be reported.

Adverse Reaction (AR)

An untoward and unintended response in a participant to an investigational medicinal product which is related to any dose administered to that participant.
The phrase "response to an investigational medicinal product" means that a causal relationship between a trial medication and an AE is at least a reasonable possibility, i.e. the relationship cannot be ruled out.
All cases judged by either the reporting medically qualified professional or the Sponsor as having a reasonable suspected causal relationship to the trial medication qualify as adverse reactions.

9.2.2 Serious adverse events (SAEs)

A serious adverse event is any untoward medical occurrence or effect that

- results in death;
- is life threatening (at the time of the event);
- requires hospitalisation or prolongation of existing inpatients' hospitalisation;
- results in persistent or significant disability or incapacity;
- is a congenital anomaly or birth defect; or
- any other important medical event that did not result in any of the outcomes listed above due to medical or surgical intervention but could have been based upon appropriate judgement by the investigator.

An elective hospital admission will not be considered as a serious adverse event.

The registration of SAEs will be limited to SAEs that result in life threatening events, requires hospitalization or death. SAEs will be reported only from the moment of inclusion until one day after the last blood taking after 365 days. The sponsor will report the SAEs through the web portal *ToetsingOnline* to the accredited METC that

approved the protocol, within 7 days of first knowledge for SAEs that result in death or are life threatening followed by a period of maximum of 8 days to complete the initial preliminary report. All other SAEs will be reported within a period of maximum 15 days after the sponsor has first knowledge of the serious adverse events.

9.2.3 Suspected unexpected serious adverse reactions (SUSARs)

Adverse reactions are all untoward and unintended responses to an investigational product related to any dose administered.
Unexpected adverse reactions are SUSARs if the following three conditions are met:
1. the event must be serious (see chapter 9.2.2);
2. there must be a certain degree of probability that the event is a harmful and an undesirable reaction to the medicinal product under investigation, regardless of the administered dose;
the adverse reaction must be unexpected, that is to say, the nature and severity of the adverse reaction are not in agreement with the product information as recorded in:
- Summary of Product Characteristics (SPC) for an authorized medicinal product;
- The sponsor will report expedited the following SUSARs through the web portal ToetsingOnline to the METC :
- SUSARs that have arisen in the clinical trial that was assessed by the METC;
- SUSARs that have arisen in other clinical trials of the same sponsor and with the same medicinal product, and that could have consequences for the safety of the subjects involved in the clinical trial that was assessed by the METC.
The remaining SUSARs are recorded in an overview list (line-listing) that will be submitted once every half year to the METC. This line-listing provides an overview of all SUSARs from the study medicine, accompanied by a brief report highlighting the main points of concern.
The expedited reporting of SUSARs through the web portal Eudravigilance or ToetsingOnline is sufficient as notification to the competent authority.
The sponsor will report expedited all SUSARs to the competent authorities in other Member States, according to the requirements of the Member States.
The expedited reporting will occur not later than 15 days after the sponsor has first knowledge of the adverse reactions. For fatal or life threatening cases the term will be maximal 7 days for a preliminary report with another 8 days for completion of the report. Principal investigators of the participating centers in this multicenter study will report SUSARs to the coordinating investigator of this study and the coordinating investigator will be responsible for report the SUSARs as described above.

Procedures for reporting Adverse Events

Solicited AEs
Participants will be asked to record local and systemic AE's for 7 days in their paper diary.
Participants are asked to fill in these AE's in a digital questionnaire 8 days after boost.
Local solicited AEs; pain, redness, warmth, swelling
Systemic solicited AEs; joint pain, fatigue, fever, chills, headache, muscle pain, nausea

Unsolicited AEs
All local and systemic AEs occurring 28 days following boost observed by the Investigator or reported by the participant.

Medically attended AEs
A medically attended AE, is defined as any adverse event for which the participant seeks medical attention either at hospital or from primary care. This explicitly excludes seeking medical attention solely for a SARS-CoV-2 test. Participants will be asked to record any medically attended AEs, either directly to the investigator or by filling in a questionnaire (every Study Visit). Medically attended AEs occurring up to 3 months post boost, will be directly solicited and reviewed at each study visit.

9.3 Annual safety report

In addition to the expedited reporting of SUSARs, the sponsor will submit, once a year throughout the clinical trial, a safety report to the accredited METC, competent authority, and competent authorities of the concerned Member States.
This safety report consists of:
- a list of all suspected (unexpected or expected) serious adverse reactions, along with an aggregated summary table of all reported serious adverse reactions, ordered by organ system, per study;
- a report concerning the safety of the subjects, consisting of a complete safety analysis and an evaluation of the balance between the efficacy and the harmfulness of the medicine under investigation.

9.4 Follow-up of adverse events

All SAEs will be followed until they have abated, or until a stable situation has been reached. Depending on the event, follow up may require additional tests or medical procedures as indicated, and/or referral to the general physician or a medical specialist. SAEs need to be reported till end of study within the Netherlands, as defined in the protocol.

9.5 [Data Safety Monitoring Board (DSMB) / Safety Committee]

If the subjects had not participated in this trial, they probably could have received the same vaccine via the national vaccination campaign at the end of this year with no

additional safety surveillance. Weekly investigator meetings will be held to discuss, among others, the different types of Aes.

10. STATISTICAL ANALYSIS

The data will be analysed as follows. *First*, the baseline characteristics of the sample will be described using descriptive statistics. Continuous variables will be presented as mean ± standard deviation in case of a normal distribution or median (interquartile range) in case of non-normal distribution. Normality of the distribution of the continuous variable will be assessed by the Shapiro-Wilk test. Categorical variables will be presented as numbers (percentage). Differences in comparing two groups or arms for continuous variables will be compared using t-test or Mann-Whitney U-test (whichever appropriate based on the normality of the variable distribution). Pearson Chi-square test or Fisher's exact test will be used to examine differences for categorical variables (depending on minimum number of 5 observations per cell, respectively). For the primary outcome, measuring the fold change (day of boost and day 28) in the antibody response after an additional boost comparing Janssen and mRNA primed HCW. The difference between the Janssen and mRNA group will be assessed using a Mann Whitney U test. For the secondary outcomes, we will use similar statistical tests as described under the baseline characteristics.The analysis will be on an intention-to-treat basis. Taking into account a possible multiple-testing problem, we will use a p-value below 0.01 as a cut off to conclude statistical significance.

10.1 Primary study parameter(s)

The primary endpoint is to determine the fold change in antibody level at day of boost and 28 days after an additional COVID-19 booster vaccination in the direct boost group, comparing Janssen with mRNA-primed HCW.

To specifically answer the key objective, we have defined two questions with the following outcome parameters:

1) *Is there an increase in antibody levels between day of boost and 28 days after boosting HCW that were initially primed with either the Janssen or an mRNA-based vaccine?*
Outcome: Level and fold change of antibodies determined by a quantitative IgG assay comparing the Janssen primed and mRNA-based primed HCW.

2) *Does booster vaccination lead to a rapid secondary recall response, indicative of immunological memory?*
Outcome: Level and fold change of antibodies and T-cell responses determined by a quantitative IgG assay and whole blood IFNγ release assay, respectively, comparing day 7 and 28 post-boost.

Depending on whether it is possible to conduct this study with a bivalent vaccine, we will add an Omicron BA.1 IgG test to the arsenal of analyses.

10.2 Secondary study parameter(s)

In addition to the primary endpoints defined above, this study will address the following secondary objectives:

1. *What is the difference in booster immunogenicity comparing a direct boost with a postponed boost?*
   Outcome: Level of antibodies and T-cell responses 7 and 28 days post boost in DB versus PPB group.

2. *What is the breadth of the immune responses after booster vaccination?*
   Outcome: PRNT against relevant variants in a random selection of study participants.

3. *What is the predictive value of immune responses on day 7 post boost?*
   Outcome: Relationship between antibodies and T-cell responses on day 7 and 28 post boost.

4. *What is the difference in reactogenicity 7 days after boost comparing the Janssen and mRNA primed HCW?*
   Outcome: Adverse events (AE) first 7 days after an additional boost between Janssen and mRNA primed HCW.

5. *Initial examination of breakthrough infections before and during study period*
   Outcome: Database of breakthrough infections in included participants based on positive PCR, self-reported positive lateral flow test, or detection of N-specific antibodies.

Exploratory objectives

Exploratory objectives (dependent on vaccine type and analyzed outside requested funding):

- Gene expression profiles associated with recall response (PAXgene tube)
- SARS-CoV-2-specific T-cell responses (PBMC, assessed by activation-induced marker assay and / or TCRbeta sequencing.

10.3 Other study parameters

N/A

10.4 Interim analysis (if applicable)

N/A

11. ETHICAL CONSIDERATIONS

11.1 Regulation statement

The study will be performed in accordance with ethical principles that have their origin in the Declaration of Helsinki (64th version, October 2013) and are consistent with the International Conference on Harmonisation (ICH), Good Clinical Practice (GCP) guidelines, applicable regulatory requirements. The Investigator must also comply with all applicable privacy directives and regulations (e.g., EU Data protection Directive 95/46/EC). The principal investigator is responsible for the proper conduct of the study at the study site.

11.2 Recruitment and consent

First, we will approach all people from the SWITCH and HCW study. If insufficient participants emerge from these studies, we will expand our search for healthcare workers in hospitals.
- Participants from SWITCH and HCW study will be approached by e-mail.

The group of persons that will be approached for this study are all Health Care Workers vaccinated once with Janssen or twice with Moderna or Pfizer and received at least one booster. Currently available vaccines have retained some degree of efficacy against all epidemiologically important SARS-CoV-2 variants that have emerged to date, with higher efficacy against hospitalization and death than against mild disease (6-11).
Results from observational studies have shown decreased effectiveness against certain variants (e.g. Omicron) and waning effectiveness over time. It is believed that revaccination will re-activate the immune memory leading to an improved protection against SARS-CoV-2.

Screening and Eligibility Assessment

Once participants express an interest in joining the trial, their e-mail address is connected to an online screening questionnaire for eligibility. This questionnaire will assess for exclusion criteria. If they pass this stage, they receive extra information (PIF).

ICH-GCP guidelines will be followed in informing the participant and obtaining consent. Before informed consent may be obtained, the participant will be given the time and opportunity to inquire about details of the trial and to decide whether or not to participate. All participants' questions about the trial will be answered before signing the informed consent form. Written informed consent of participants is required before enrolment in the trial and before any study related procedure will take place.

The content of the participant information letter, informed consent form and any other written information to be provided to participants will be in compliance with ICH-GCP, GDPR and other applicable regulations and will be approved by the ethics committee in advance of use. The participant information letter, informed consent form and any other written information to be provided to participants will be revised whenever important new information becomes available that may be relevant to the participant's consent. Any substantially revised informed consent form and written information will be approved by the ethics committee in advance of use. The participant will be informed in a timely

manner if new information becomes available that might be relevant to the participant's willingness to continue participation in the trial.

11.3 Objection by minors or incapacitated subjects (if applicable)

N/A

11.4 Benefits and risks assessment, group relatedness

1. If RIVM decides that a bivalent vaccine should be applied, we believe that modifying strain composition of COVID-19 vaccines would be more effective against circulating and potentially merging virus variants and would have a more benefit-risk balance than currently available vaccines (12), like retaining neutralization for ancestral SARS-CoV-2, a stronger immune response against current variants, broader cross-neutralization against future variants en extend durability of protection (13).

2. The combination of different vaccines (homologous and heterologous vaccination regimens) lead to sufficient protection and is safe as seen in previous studies (2, 14-17).

3. The occurrence of side effects is similar for both homologous and heterologous vaccination regimens (2, 14-18).

4. It is important to state that the vaccines for this study will NOT be retrieved from a stock reserved for patients or other persons. The vaccines will be supplied by the RIVM after approval by ZonMw and VWS.

5. Each participant will receive a measurement of their immune response to SARS-CoV-2 and will know their immune titer approximately 6 weeks after the booster. This description indicates whether you are adequately protected or not. If someone is not properly protected, we will provide sufficient protection by administering an extra vaccine.

Conclusion

Based on the considerations mentioned above and weighing both the risks and the benefits of this trial, we conclude that there is a positive risk-benefit analysis to proceed with this trial.

11.5 Compensation for injury

The sponsor/investigator has a liability insurance which is in accordance with article 7 of the WMO. The sponsor (also) has an insurance which is in accordance with the legal requirements in the Netherlands (Article 7 WMO). This insurance provides cover for damage to research subjects through injury or death caused by the study. The insurance applies to the damage that becomes apparent during the study or within 4 years after the end of the study. The sponsor/investigator has a liability insurance which is in accordance with article 7 of the WMO. The sponsor (also) has an insurance which is in accordance with the legal requirements in the Netherlands (Article 7 WMO). This insurance provides cover for damage to research subjects through injury or death caused by the study. The insurance applies to the damage that becomes apparent during the study or within 4 years after the end of the study.

11.6 Incentives (if applicable)

N/A

12. ADMINISTRATIVE ASPECTS, MONITORING AND PUBLICATION

12.1 Handling and storage of data and documents

Data and documents will be controlled and processed conform the EU General Data Protection Regulation (GDPR) and the Dutch Act on Implementation of the General Data Protection Regulation. (in Dutch: Uitvoeringswet AVG, UAVG).

i. Confidentiality of the data

By signing this protocol, the investigator affirms to the Sponsor that information furnished to the investigator will be and such information will be divulged to the institutional review board, ethics review committee (IRB/ERC) or similar or expert committee; affiliated institution and employees, only under an appropriate understanding of confidentiality with such board or committee, affiliated institution and employees. Data generated by this trial will be considered confidential by the investigator, except to the extent that it is included in a publication as provided in the Publications section of this protocol.

ii. Confidentiality of subject records

Prior to enrollment in the study and prior to any study related procedure, the participant must personally sign and date the informed consent form.
Each participant will be given a unique sequential study subject number not based on the participant's initials or birthdate. The key to the participant study number is safeguarded by the local investigator.
Data will be collected using an eCRF (electronic case report form) designed for this study. According to ICH guidelines for Good Clinical Practice, the monitoring team must check the specific CRF entries against the source documents, as specified in the study specific monitoring plan. The Informed Consent Form will include a statement by which the participant allows the Sponsor's duly authorized personnel, the ethics review committee (IRB/ERC) or similar or expert committee, and the regulatory authorities to have direct access to original medical records which support the data on the CRFs (e.g. participant's medical file).
This personnel, bound by professional secrecy, must maintain the confidentiality of all personal identity or personal medical information, according to confidentiality and personal data protection rules and in compliance with all applicable privacy laws, rules, and regulations.

iii. Data Management

The investigator or qualified designee is responsible for recording and verifying the accuracy of subject data. By signing this protocol, the investigator acknowledges that his/her electronic signature is the legally binding equivalent of a written signature. By entering his/her electronic signature, the investigator confirms that all recorded data have been verified as accurate.
Data will be stored for 25 years.

iv. Biological samples

Biological samples will only be stored for the purpose of additional research if the participant has given consent. Material that is not used for current translational research will be stored for up to 15 years after end of study. If no informed consent was obtained, samples will be destroyed after the participant has completed all protocol treatment and procedures. Storage of biological samples on site is subject to the site's guidelines.

12.2 Monitoring and Quality Assurance

On-site monitoring will take place according to the NFU (Nederlandse Federatie van Universitaire Medisch Centra)-guideline “Kwaliteitsborging van mensgebonden onderzoek 2019” by the appointed monitor. This study is classified as negligible risk because vaccination is standard of care. Monitoring will take place to assure the quality and validity of the research data. The monitor will perform source data verification on the research data by comparing the data entered into the CRF with the available source documentation and other available documents. Source documents are defined as the patient's hospital medical records, clinician notes, laboratory print outs, digital and hard copies of imaging, memos, electronic data etc.

The monitor will verify the following items: Patient flow (inclusion speed and dropout rate); Informed consent forms (presence, dates, signatures); Informed consent process, Trial Master File and Investigator Files (presence of all documents), in-/exclusion criteria (using source documents). After each control the monitor will send a written report to the sponsor (including a summary; quality assessment; summary of findings, deviations and shortcomings; possible solutions to warrant compliance with the protocol; final conclusion).

12.3 Amendments

Amendments are changes made to the research after a favourable opinion by the accredited METC has been given. All amendments will be notified to the METC that gave a favourable opinion.

All substantial amendments will be notified to the METC and to the competent authority.

Non-substantial amendments will not be notified to the accredited METC and the competent authority, but will be recorded and filed by the sponsor.

12.4 Annual progress report

The sponsor/investigator will submit a summary of the progress of the trial to the accredited METC once a year. Information will be provided on the date of inclusion of the first subject, numbers of subjects included and numbers of subjects that have completed the trial, serious adverse events/ serious adverse reactions, other problems, and amendments.

12.5 Temporary halt and (prematurely) end of study report

The investigator/sponsor will notify the accredited METC of the end of the study within a period of 8 weeks. The end of the study is defined as the last participant's last visit.

The sponsor will notify the METC immediately of a temporary halt of the study, including the reason of such an action.

In case the study is ended prematurely, the sponsor will notify the accredited METC within 15 days, including the reasons for the premature termination.

Within one year after the end of the study, the investigator/sponsor will submit a final study report with the results of the study, including any publications/abstracts of the study, to the accredited METC.

12.6 Public disclosure and publication policy

Trial results will be submitted for publication in a peer reviewed scientific journal regardless of the outcome of the trial. Results may not be published if the trial was terminated prematurely and/ or did not yield sufficient data for a publication.

13. STRUCTURED RISK - BENEFIT ANALYSIS

13.1 Potential issues of concern

13.2 Synthesis

Benefits and risks assessment, group relatedness

1. The group of persons that will be approached for this study are all Health Care Workers vaccinated once with Janssen or twice with Moderna or Pfizer and received at least one booster. Currently available vaccines have retained some degree of efficacy against all epidemiologically important SARS-CoV-2 variants that have emerged to date, with higher efficacy against hospitalization and death than against mild disease (6-11).
   Results from observational studies have shown decreased effectiveness against certain variants (e.g. Omicron) and waning effectiveness over time. It is believed that revaccination will re-activate the immune memory leading to an improved protection against SARS-CoV-2.

2. If RIVM decides that a bivalent vaccine should be applied, we believe that modifying strain composition of COVID-19 vaccines would be more effective against circulating and potentially merging virus variants and would have a more benefit-risk balance than currently available vaccines (12), like retaining neutralization for ancestral SARS-CoV-2, a stronger immune response against current variants, broader cross-neutralization against future variants en extend durability of protection (13).

3. The combination of different vaccines (homologous and heterologous vaccination regimens) lead to sufficient protection and is safe as seen in previous studies (2, 14-17).

4. The occurrence of side effects is similar for both homologous and heterologous vaccination regimens (2, 14-18).

5. It is important to state that the vaccines for this study will NOT be retrieved from a stock reserved for patients or other persons. The vaccines will be supplied by the RIVM after approval by ZonMw and VWS.

6. Each participant will receive a measurement of their immune response to SARS-CoV-2 and will know their immune titer approximately 6 weeks after the booster. This description indicates whether you are adequately protected or not. If someone is not properly protected, we will provide sufficient protection by administering an extra vaccine.

Conclusion

Based on the considerations mentioned above and weighing both the risks and the benefits of this trial, we conclude that there is a positive risk-benefit analysis to proceed with this trial.

14. REFERENCES

1. Sablerolles RSG, Goorhuis A, GeurtsvanKessel CH, de Vries RD, Huckriede ALW, Koopmans MPG, et al. Heterologous Ad26.COV2.S Prime and mRNA-Based Boost COVID-19 Vaccination Regimens: The SWITCH Trial Protocol. Front Immunol. 2021;12:753319.
2. Sablerolles RSG, Rietdijk WJR, Goorhuis A, Postma DF, Visser LG, Geers D, et al. Immunogenicity and Reactogenicity of Vaccine Boosters after Ad26.COV2.S Priming. N Engl J Med. 2022.
3. Sablerolles RSG, Rietdijk WJR, Goorhuis A, Postma DF, Visser LG, Schmitz KS, et al. Durability of immune responses after boosting in Ad26.COV2.S-primed healthcare workers. Clinical Infectious Diseases. 2022.
4. Geers D, Shamier MC, Bogers S, den Hartog G, Gommers L, Nieuwkoop NN, et al. SARS-CoV-2 variants of concern partially escape humoral but not T cell responses in COVID-19 convalescent donors and vaccine recipients. Science Immunology. 2021;6(59):eabj1750.
5. GeurtsvanKessel CH, Geers D, Schmitz KS, Mykytyn AZ, Lamers MM, Bogers S, et al. Divergent SARS CoV-2 Omicron-reactive T- and B cell responses in COVID-19 vaccine recipients. Sci Immunol. 2022:eabo2202.
6. Tseng HF, Ackerson BK, Luo Y, Sy LS, Talarico CA, Tian Y, et al. Effectiveness of mRNA-1273 against SARS-CoV-2 Omicron and Delta variants. Nature Medicine. 2022;28(5):1063-71.
7. Andrews N, Stowe J, Kirsebom F, Toffa S, Rickeard T, Gallagher E, et al. Covid-19 Vaccine Effectiveness against the Omicron (B.1.1.529) Variant. New England Journal of Medicine. 2022;386(16):1532-46.
8. Stowe J, Andrews N, Kirsebom F, Ramsay M, Bernal JL. Effectiveness of COVID-19 vaccines against Omicron and Delta hospitalisation: test negative case-control study. medRxiv. 2022.
9. Chemaitelly H, Ayoub HH, AlMukdad S, Coyle P, Tang P, Yassine HM, et al. Duration of mRNA vaccine protection against SARS-CoV-2 Omicron BA.1 and BA.2 subvariants in Qatar. medRxiv. 2022:2022.03.13.22272308.
10. Bar-On YM, Goldberg Y, Mandel M, Bodenheimer O, Amir O, Freedman L, et al. Protection by 4th dose of BNT162b2 against Omicron in Israel. medRxiv. 2022:2022.02.01.22270232.
11. Associated Hospitalizations Among Adults During SARS-CoV-2 Delta and Omicron Variant Predominance, by Race/Ethnicity and Vaccination Status [press release]. MMWR Morb Mortal Wkly Rep 2022;71; 466-4732022.
12. FDA Briefing Document - Vaccines and Related Biological Products Advisory Committee Meeting - SARS-CoV-2 strain composition of COVID-19 vaccines 2022 [Available from: https://www.fda.gov/media/159452/download.
13. mRNA-1273.214 - Moderna COVID-19 Investigational Bivalent Vaccine (Original + Omicron) - Moderna, Inc. - Vaccines and Related Biological Products Advisory Committee June 28, 2022 2022 [Available from: https://www.fda.gov/media/159492/download.
14. Borobia AM, Carcas AJ, Pérez-Olmeda M, Castaño L, Bertran MJ, García-Pérez J, et al. Immunogenicity and reactogenicity of BNT162b2 booster in ChAdOx1-S-primed participants (CombiVacS): a multicentre, open-label, randomised, controlled, phase 2 trial. The Lancet. 2021;398(10295):121-30.
15. Liu X, Shaw RH, Stuart ASV, Greenland M, Aley PK, Andrews NJ, et al. Safety and immunogenicity of heterologous versus homologous prime-boost schedules with an adenoviral vectored and mRNA COVID-19 vaccine (Com-COV): a single-blind, randomised, non-inferiority trial. The Lancet. 2021;398(10303):856-69.
16. Hillus D, Schwarz T, Tober-Lau P, Vanshylla K, Hastor H, Thibeault C, et al. Safety, reactogenicity, and immunogenicity of homologous and heterologous prime-boost immunisation with ChAdOx1 nCoV-19 and BNT162b2: a prospective cohort study Lancet Respir Med. 2021.

17. Atmar RL, Lyke KE, Deming ME, Jackson LA, Branche AR, El Sahly HM, et al. Homologous and Heterologous Covid-19 Booster Vaccinations. N Engl J Med. 2022;386(11):1046-57.
18. Shaw RH, Stuart A, Greenland M, Liu X, Van-Tam JSN, Snape MD. Heterologous prime-boost COVID-19 vaccination: initial reactogenicity data. The Lancet. 2021;397(10289):2043-6.
